# Editorial: Functional feed additives and intestinal health in aquatic animals

**DOI:** 10.3389/fphys.2024.1385046

**Published:** 2024-03-13

**Authors:** Gang Yang, Zhen Zhang, Vikash Kumar

**Affiliations:** ^1^ Department of Fisheries Science, School of Life Science, Nanchang University, Nanchang, China; ^2^ Key Laboratory for Feed Biotechnology of the Ministry of Agriculture and Rural Affairs, Institute of Feed Research, Chinese Academy of Agricultural Sciences, Beijing, China; ^3^ Aquatic Environmental Biotechnology and Nanotechnology (AEBN) Division, ICAR-Central Inland Fisheries Research Institute (CIFRI), Kolkata, India

**Keywords:** feed additives, immunity, intestinal inflammation, antioxidant capacity, intestinal microbiota, metabolism

The rapid expansion and intensification of aquaculture have been accompanied by various intestinal disease outbreaks and a drastic increase in mortality, especially when fish meal and oil in feed were replaced by plant source raw materials in large quantities (Glencross et al., 2020). Previous studies have identified dietary supplementation of functional feed additives as one of the alternative strategies and therapeutic interventions to prevent intestinal dysfunction that benefits the healthy growth of aquatic animals simultaneously (Dawood et al., 2018; Hossain et al., 2024). Except for digestion and absorption functions, the intestine also serves as the largest immunity organ and performs as the first barrier of defense between the organism and pathogens, and thus, it is crucial for fish growth and physiological functions. Therefore, it is utmost importance to investigate different functional additives to improve the intestinal health of aquatic animals.

The goal of this Research Topic is to focus on the effects of functional feed additives on intestinal health of aquatic animals by studying their benefits on the intestinal physiological state and underlying mechanisms. The areas covered include: 1) Feed additives and intestinal immunity; 2) Anti-inflammatory mechanism; 3) Antioxidant stress regulation; 4) The relationship between feed additives and nutrient metabolism; and 5) The modulating of the intestinal microbiome by feed additives.

In past decades, plant source raw materials have been widely used, and their proportion in aquatic feed have become higher and higher due to the shortage of fishery resources and their expensive price (Macusi et al., 2023). When basic feed resources become scarce, assessing alternative replacements the from natural environment becomes crucial to sustaining the fast growth of aquaculture, which supports billions of people by providing high-quality animal protein (Valenti et al., 2018). The studies included in this Research Topic are mainly focused on the effects of different diets derived from wheat, soybean oil, corn starch, and frass from black soldier on the growth, feed utilization, immune response, and welfare of aqua-farmed animals. Zhang et al. demonstrated the positive role of replacing of fish meal and soy protein with wheat gluten on growth performance, feed utilization, and nutrient digestibility and retention in Japanese seabass (*Lateolabrax japonicus*). Zuo et al. evaluated the effects of different carbohydrate-to-lipid ratios (C/L) on growth and energy utilization and their mechanism in Chinese mitten crab (*Eriocheir sinensis*), and their results revealed that the optimal dietary C/L of 3.59 was beneficial for growth performance and carbohydrate and lipid metabolism. In this Research Topic, an article by Sankappa et al. highlighted that dietary inclusion of frass from black soldier fly larvae improved the immunity of the fish by activating the innate and adaptive immunity of channel catfish (*Ictalurus punctatus*).

Functional feed additives have been widely used in aquaculture due to their positive effects on aquatic animals (Vijayaram et al., 2022). An inclusion of selenium as a functional feed additive by Li et al. demonstrated its antioxidant effects in aquatic animals. The supplementation of 0.01%–0.02% fulvic acid in diet was identified to be beneficial for growth performance, digestive ability, and intestinal function of large yellow croaker (*Larimichthys crocea*) larvae through the inhibition of intestinal inflammation (Zhang et al.). Yang et al. highlighted that dietary supplementation of 1%–2% fermented *Astragalus membranaceus* could improve intestinal and hepatic morphology and regulate intestinal microbiota together with a significant enhancement in the growth performance of juvenile tiger grouper (*Epinephelus fuscoguttatus*). Pelusio et al. and Sun et al. reported that supplementation of yeast extract improved the growth, digestibility, intestinal histology, antioxidant capacity, and immune response and modulated the intestinal microbiota in fish positively. Moreover, Pelusio et al. pinpointed that nucleotides and nucleic acids extracted from yeast can be an excellent fish meal replacer. Li et al. investigated the application of fucoidan in juvenile common carp (*Cyprinus carpio*) and highlighted its positive effects on growth performance, immunity, antioxidant ability, digestive enzyme activity, and hepatic morphology. Bera et al. reported that the intestinal microbiome of *Oreochromis niloticus* could be optimized through nutritional interventions with Aloe vera extract polysaccharide, thereby improving the performance in commercial fish.

The underlying regulatory mechanism of functional feed additives improving the intestinal health of aquatic animal is far from being understood. The studies included in this Research Topic present the effects of wheat gluten, the variation in the ratios of dietary carbohydrates to lipids, and frass from black soldier fly larvae on organic physiology and highlighted the latest discoveries and advances of functional feed additives in aquatic animals. By compiling these 10 articles, we provide a base of knowledge for both researchers and professionals in the field of aquaculture for the better understanding of the application of functional feed additives in aquaculture farming. Hence, these articles will hopefully upgrade our understanding on the contribution of different types of diets and functional feed additives isolated from various natural sources on the wellbeing of aquatic animals.
